# In Vitro Cytotoxic Effects of Ferruginol Analogues in Sk-MEL28 Human Melanoma Cells

**DOI:** 10.3390/ijms242216322

**Published:** 2023-11-14

**Authors:** Luying Shao, Miguel A. González-Cardenete, Jose M. Prieto-Garcia

**Affiliations:** 1 School of Pharmacy, University College London, London WC1E 6HX, UK; luying.shao.12@ucl.ac.uk; 2 Instituto de Tecnología Química (UPV-CSIC), Universitat Politècnica de Valencia-Consejo Superior de Investigaciones Científicas, Avda de los Naranjos s/n, 46022 Valencia, Spain; migoncar@itq.upv.es; 3 Centre for Natural Products Discovery, School of Pharmacy and Biomolecular Sciences, Liverpool John Moores University, Liverpool L3 5UX, UK

**Keywords:** diterpenes, ferruginol, apoptosis, melanoma, migration, caspases

## Abstract

Ferruginol is a promising abietane-type antitumor diterpene able to induce apoptosis in SK-Mel-28 human malignant melanoma. We aim to increase this activity by testing the effect of a small library of ferruginol analogues. After a screening of their antiproliferative activity (SRB staining, 48 h) on SK-Mel-28 cells the analogue 18-aminoferruginol (GI50 ≈ 10 µM) was further selected for mechanistic studies including induction of apoptosis (DAPI staining, *p* < 0.001), changes in cell morphology associated with the treatment (cell shrinkage and membrane blebbing), induction of caspase-3/7 activity (2.5 at 48 h, 6.5 at 72 h; *p* < 0.0001), changes in the mitochondrial membrane potential (not significant) and in vitro effects on cell migration and cell invasion (Transwell assays, not significant). The results were compared to those of the parent molecule (ferruginol, GI50 ≈ 50 µM, depolarisation of mitochondrial membrane *p* < 0.01 at 72 h; no caspases 3/7 activation) and paclitaxel (GI50 ≈ 10 nM; caspases 3/7 activation *p* < 0.0001) as a reference drug. Computational studies of the antiproliferative activity of 18-aminoferruginol show a consistent improvement in the activity over ferruginol across a vast majority of cancer cells in the NCI60 panel. In conclusion, we demonstrate here that the derivatisation of ferruginol into 18-aminoferruginol increases its antiproliferative activity five times in SK-MEL-28 cells and changes the apoptotic mechanism of its parent molecule, ferruginol.

## 1. Introduction

Natural products are a continuous source of cytotoxic compounds that lead to cancer treatment [1]. Malignant melanoma, the most aggressive skin cancer, accounts for about 3% of malignant tumour cases. Its incidence is rising worldwide, becoming resistant to therapeutic agents [2].

Our recent review highlighted the therapeutic potential of diterpenes against melanoma [3]. This class of phytochemicals exhibits high structural diversity and potent biological activities due to their unique carbon skeletons. They have traditionally been a source for developing new anticancer agents. Taxol, the first “blockbuster diterpene,” was initially selected for its activity on murine melanoma cells [4]. The most recent discovery in this class of metabolites is ingenol mebutate, indicated for the chemoprevention of melanoma in patients with actinic keratosis [3].

Abietane-type diterpenes, characterised by a tricyclic ring system, include some compounds that show significant cytotoxicity against melanoma cells. We have extensively reviewed their chemistry and biology in a previous work [5]. Examples include carnosol and carnosic acid, two well-known phenolic diterpenes from *Rosmarinus officinalis* L. extracts [6,7], and other lesser-known diterpenes such as Parvifloron D from *Plectranthus ecklonii* Benth [8], 11,12,16-trihydroxy-2-oxo-5-methyl-10-demethyl-abieta-1[10],6,8,11,13-pentene from *Premna serratifolia* L. [9] and 7a-acetoxyroyleanone and horminone from roots of *Peltodon longipes* A. St. Hill. Ex Benth [10]. All these plants belong to the same botanical family (Lamiaceae). Another abundant source of these diterpenes is the rosin from coniferous species such as pine trees. Abietic acid and dehydroabietic acid, the primary diterpenic acids found in *Pinus* rosin, have potential as anticancer agents. They have demonstrated significant growth inhibitory activity against various human cancer cells, including breast, ovary, prostate, colon, liver, lung and cervix lines [11].

**Figure 1:**
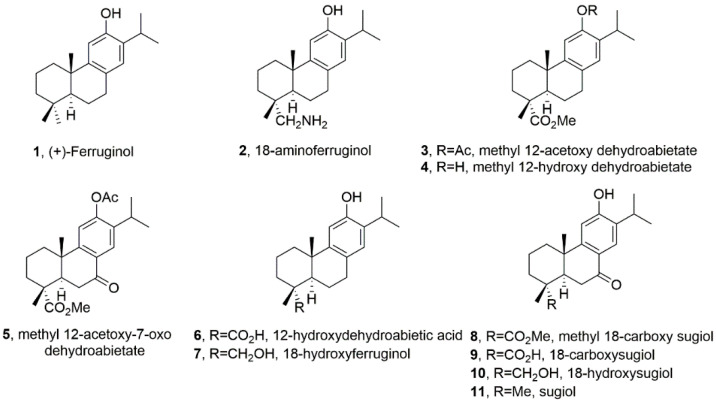
Chemical structures of ferruginol and analogues are studied in this work.

Ferruginol (**1**, Figure 1), another promising abietane-type antitumor diterpene with a characteristic phenolic ring, is found in the Lamiaceae and Cupressaceae families [12]. It has shown significant cytotoxic effects on cancer cells, including gastric, prostate, lung, cervical, breast and colon cancer cells, leukaemia and melanoma cells in vitro [13]. When administered intraperitoneally, it inhibited tumour growth in mice CL1-5 xenografts [14]. Sugiol, the C7-oxidized derivative of ferruginol (**11**, Figure 1), has also demonstrated in vitro cytotoxic activity against human pancreatic and melanoma tumour cell models and in vivo antitumor properties in a prostate DU145 mouse xenograft model [15]. Ferruginol induces apoptosis in SK-Mel-28 human malignant melanoma cells using P-p38 and NF-κB mediation [16]. 

These antecedents prompted the creation of a library of semisynthetic ferruginol derivatives (Figure 1) by one of the authors (M. Gonzalez-Cardenete) and their collaborative screening against a panel of representative human cancer cells (SUM149, MDA-MB231, T47D and MCF07A549, HBL-100, HeLa, SW1573, T-47D, WiDr, NALM-06, KOPN-8, SUP-B15, UoCB1 and BCR-ABL), and also parasites (*Leishmania* sp.) and viruses (Zika, Dengue, Herpes simplex type 1 and Chikungunya) with promising results [17,18,19].

In light of the above-discussed precedents, we aim to expand our understanding of the effect of a library of ferruginol analogues (Figure 1) on human SK-MEL-28 melanoma cells.

## 2. Results

### 2.1. In Vitro Antiproliferative Activity of Ferruginol and Analogues 

Cell proliferation is a critical step in determining the effectiveness of cytotoxic small molecules [20]. We here chose the Sulforhodamine B (SRB) staining assay, developed and utilised by the US National Cancer Institute, as it is considered the gold standard for this screening method [21].

**Figure 2:**
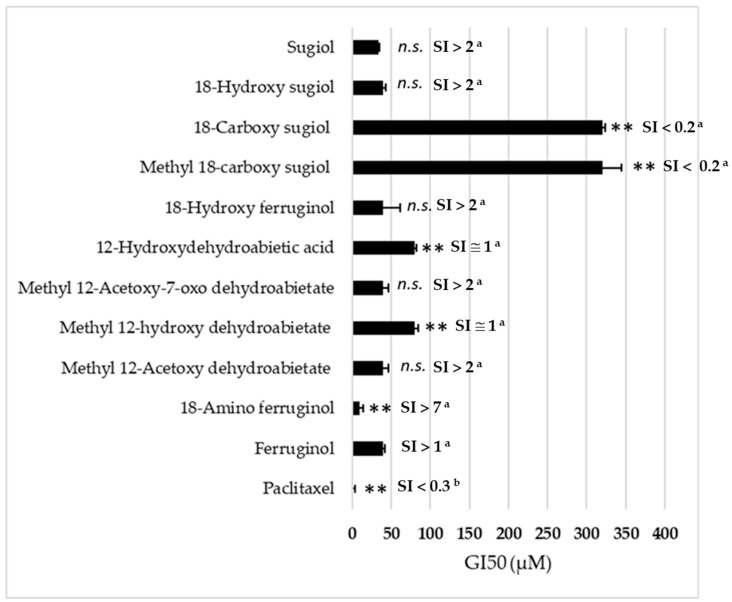
Antiproliferative activity (GI_50_, µM) in the SRB assay (48 h) of ferruginol analogues in SK-MEL-28 cells. Mean ± SD (*n* = 3). (**) *p* < 0.01; (*n.s.*) not significative. *p*-values were calculated by one-way ANOVA followed by Dunnet’s test with ferruginol (parent molecule) as control. Paclitaxel acts as a reference cytotoxic drug. SI = Selectivity Index (^a^) vs. BJ cells [17];(^b^) vs. MCR-5 cells [22].

Figure 2 presents the 50% growth inhibitory concentration (GI50, µM) of all ferruginol analogues tested on SK-MEL-28 cells in the SRB assay for 48 h. In our experiments, we used paclitaxel as a positive reference. Its GI50 was approximately 10 nM, an order of magnitude lower than the ferruginol derivatives.

**Figure 3:**
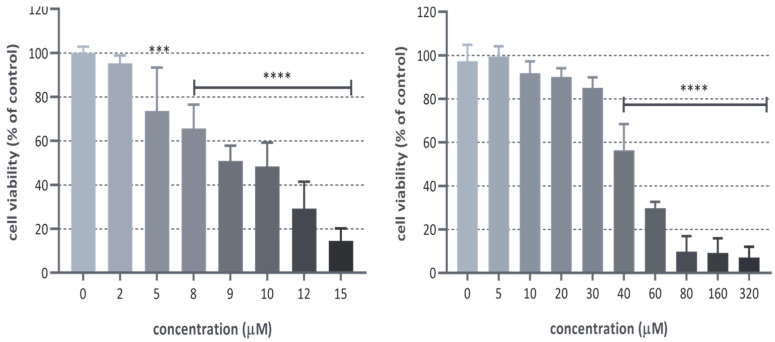
SK-MEL-28 proliferation after treatment with 18-aminoferruginol (left bar graph) and ferruginol (right bar graph) at different concentrations using the SRB assay (48 h). Mean ± SD (*n* = 4), (***) *p* < 0.001; (****) *p* < 0.0001 *p*-values were calculated by one-way ANOVA followed by Dunnet’s test, using plain solvent (DMSO) as control.

The results indicate that the introduction of an amino group in position 18 is the only chemical modification that enhances the antiproliferative activity of (+)-ferruginol (1) (GI50 = 47.5 µM). This modification results in a compound (**2**), termed 18-aminoferruginol, which exhibits approximately five times greater activity (GI50 = 9.8 µM). This activity is not significantly different from paclitaxel, the reference drug used in this study. Figure 3 illustrates the range of their antiproliferative effects.

These results must be compared with the antiproliferative effects of ferruginol and 18-aminoferruginol in normal cells. Fibroblasts are normal human foreskin cells utilised to test for general cytotoxicity. These cells are typically sensitive to compounds that induce necrosis or apoptosis and serve to calculate a selectivity index (Table 1). The results show a more favourable cytotoxicity profile for 18-aminoferruginol when compared to its parent molecule, ferruginol.

### 2.2. Changes in Cell Morphology Associated with the Treatment Point Out to the Induction of Apoptosis 

**Figure 4:**
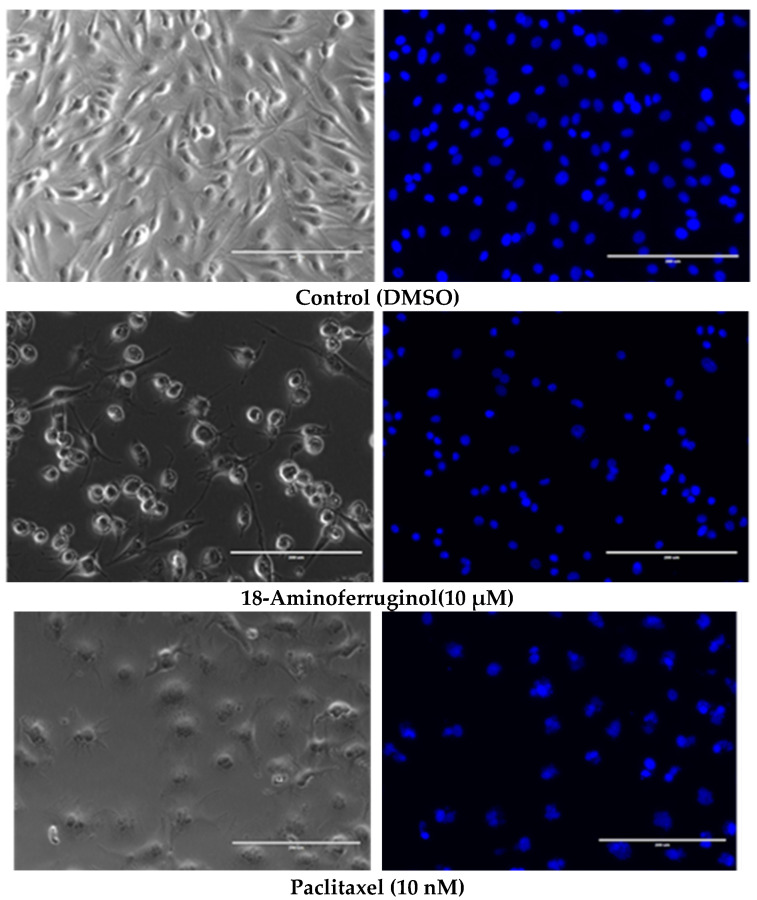
Representative light contrast microscopy (**left**) and fluorescence DAPI (**right**) 20× images of living SK-MEL-28 cells after treatment with 18-Aminoferruginol (48 h). Paclitaxel acts as a reference drug (48 h). The images were processed using ImageJ’s open-access software, (v. 1.53k) using three pictures per replicate.

**Figure 5:**
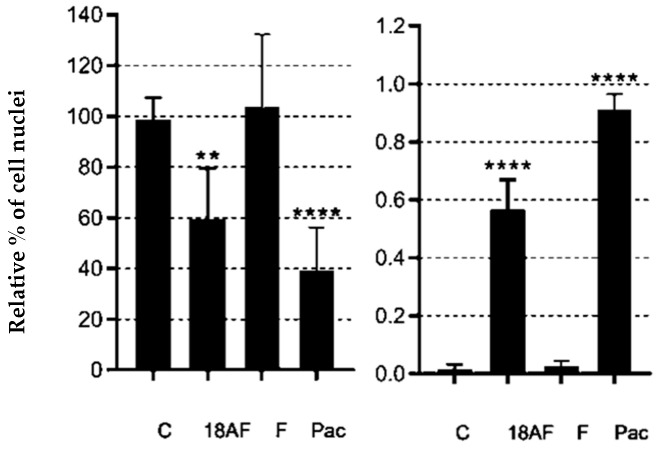
Quantification of DAPI staining after treatments with ferruginol (F) and 18-aminoferruginol (1). On the left is the count of the total number of nuclei normalised to untreated controls (C); On the right is the count of the total number of fragmented nuclei over the total number of nuclei. Bars show mean and standard errors of an average of 3 fields per condition from 3 separate plates, i.e., *n* = 9 fields per condition. *p*-values are calculated by one-way ANOVA followed by Dunnet’s test, (**) *p* < 0.01 and (****) *p* < 0.0001. Paclitaxel (Pac) acts as a reference drug.

To understand the antiproliferative activity of 18-Aminoferruginol, we evaluated cell viability and apoptosis qualitatively (Figure 4) and quantitatively (Figure 5). We used phase contrast microscopy (Figure 4, right column) and 4′,6-diamidino-2-phenylindole (DAPI) (Figure 4, left column) staining to confirm the cytotoxicity of the compounds to melanoma cells. Phase contrast images (Figure 5, left column) reveal that untreated cells grew to near confluence and spread regularly on the slide. Treated cells exhibited several apoptotic features, such as cell shrinkage and membrane blebbing over the incubation period.

After 48 h of exposure treatments, the percentage of blue-stained cells decreased compared to the control. We quantitatively evaluated the images by counting the total number of nuclei and normalising them to untreated controls. We also counted the total number of fragmented nuclei. This decrease indicates that both compounds promoted cell death (Figure 5). 

### 2.3. Discerning Early and Late Events of Intrinsic Apoptosis 

**Figure 6:**
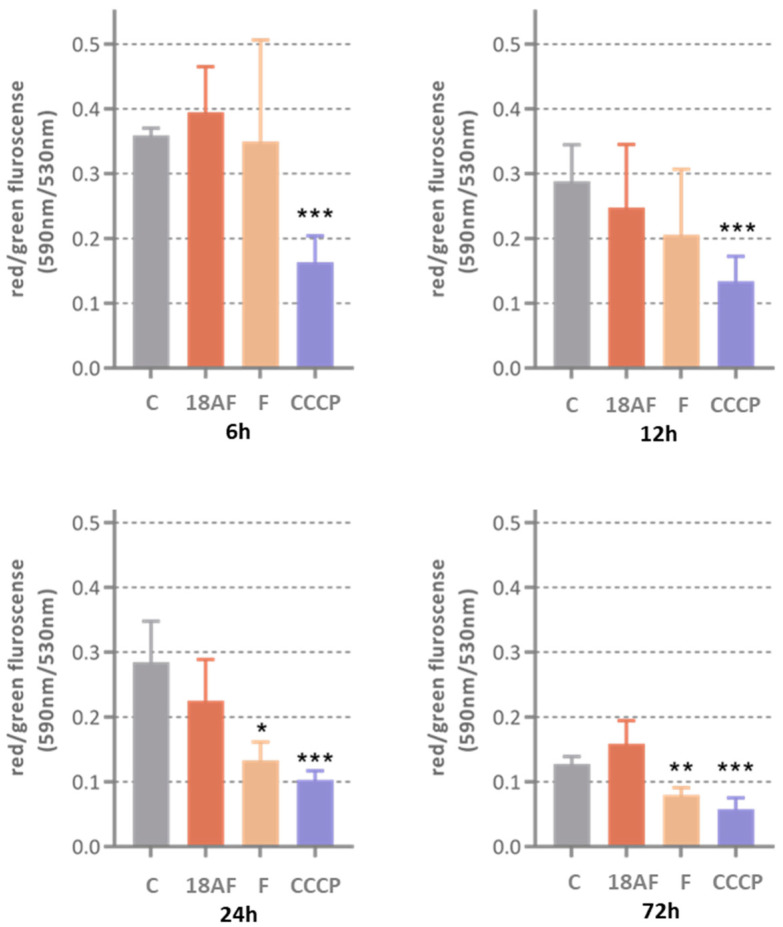
The effect of ferruginol (F) and 18-aminoferruginol (18AF) at 10 µM on mitochondrial membrane potential (10 µM) at various treatment times (6 h, 12 h, 24 h and 72 h). Bars show Mean ± SD (*n* = 4), *p*-values were determined via one-way ANOVA followed by Dunnet’s test, (*) *p* < 0.05; (**) *p* < 0.01; (***) *p* < 0.001. CCCP acts as the positive control. (C) Control (Solvent, DMSO).

**Figure 7:**
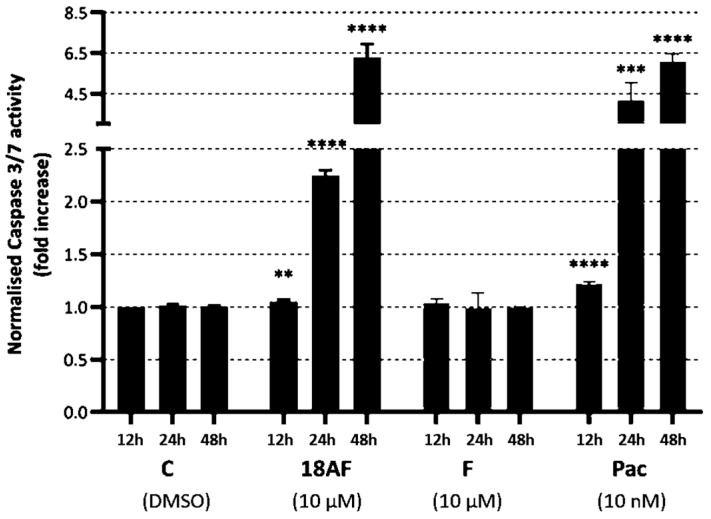
The effect of ferruginol (F) and 18-aminoferruginol (18AF) at 10 µM on caspase-3/7 activity in SK-MEL-28 cells (12, 24 and 48 h). Bars represent Mean ± SD (*n* = 4), (**) *p* < 0.01; (***) *p* < 0.001; (****) *p* < 0.0001. *p*-values were determined via one-way ANOVA followed by Dunnet’s test. Paclitaxel (Pac) acts as a reference drug. (C) Control (Solvent, DMSO).

The extrinsic and intrinsic apoptosis pathways converge at the activation of caspase-3 [23]. The loss of mitochondrial membrane potential is known to precede apoptosis and chemical hypoxia-induced necrosis [24]. To understand the mechanisms behind the apoptotic features observed in our microscopy studies, we examined the effects of ferruginol and 18-aminoferruginol on mitochondrial membrane potential (Figure 6) and caspase-3/7 activity (Figure 7), which serve as markers for early and late phase apoptosis, respectively [25].

Ferruginol (10 µM) caused depolarisation of mitochondrial membranes after a 24 h treatment, an effect that persisted up to 72 h. However, this did not lead to significant activation of caspases 3/7 after three days of treatment. The reference compound CCCP (2 µM) consistently depolarised SK-MEL-28 cells from 6 h onwards, confirming the model’s sensitivity to this effect.

In contrast, 18-aminoferruginol (10 µM) did not cause depolarisation of the mitochondrial membranes. Unlike ferruginol, it promoted caspase-3/7 activity at all time points (12, 24 and 48 h), similar to the effects of the reference drug paclitaxel (10 nM).

### 2.4. Migration Assay and Invasion Assays

While chemotherapy drugs typically target cancer cell viability, this does not prevent the metastasis of surviving cancer cells [26]. Therefore, it is crucial to determine whether cytotoxic agents can also inhibit cancer cell motility, a key factor for success in advanced stages of cancer [27].

Cell motility encompasses two distinct concepts in experimental biology: migration and invasion. Migration refers to the directed movement of cells on a substrate, such as basal membranes, extracellular matrix (ECM) fibres, or plastic plates. In contrast, invasion involves the movement of cells through a 3D matrix, requiring a more complex set of cellular processes that include restructuring the surrounding 3D environment [28].

The ability to migrate is a prerequisite for invasion; a cell can migrate without invading but not without migrating [29]. Several cell migration assays are available to evaluate the anti-metastatic activity of drugs, including 2D assays (such as wound healing/scratch assays) and 3D assays (such as transwell assays and Boyden chamber assays).

**Figure 8:**
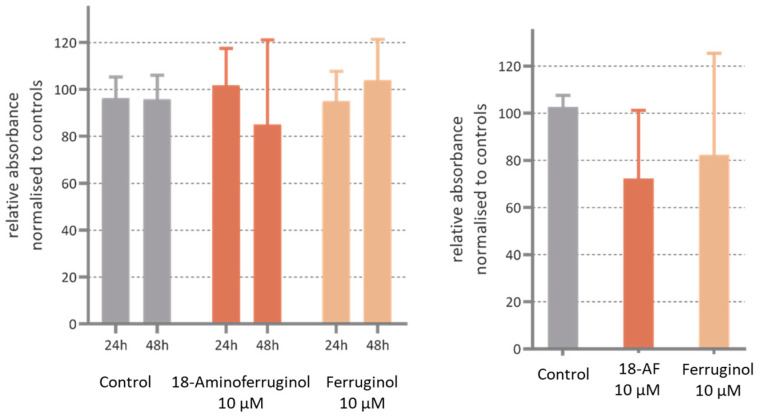
3D cell invasion activity in SK-MEL-28 cells incubated with ferruginol and 18-aminoferruginol (18-AF) for 48 h post-treatment. The *Y* axis shows the invasion ability of cells relative to the Control (Solvent) in % absorbance. The bars represent Mean ± SD (*n* = 4); *p*-values were determined via one-way ANOVA followed by Dunnet’s test (all *p* > 0.05).

Our results with antiproliferative concentrations of either ferruginol and its derivative compound 2 (18-aminoferruginol) show that they reduced the motility, although in a non-significant manner. (Figure 8). In our study, we conducted an in vitro migration assay using plain transwell assays and then coated them with an appropriate matrix to simulate cell migration/invasion (refer to Section 4.7 for details). These protocols, together with the study of proliferation using the SRB staining technique, are considered conventional label-based endpoint methods of choice [30]. However, they compromise in two aspects: real-time monitoring [30] and a realistic 3D environment [31]. This may contribute to experimental variation and challenges in identifying and validating new cancer drug targets using these cellular assays [32]. There is a possibility that with improved techniques [33], such as 3D-microfluidics [34] using a wider range of concentrations (MNTC to GI50), the errors may be reduced and significance reached.

## 3. Discussion

### 3.1. On the Ferruginol Derivatives Structure-Antiproliferative Activity Relationship

We can deduce the structure-activity relationship (SAR) of compounds **1**–**11** (Figure 1) from the data obtained in the SK-MEL-28 cell line (Figure 2). Most compounds exhibited less activity than the parent molecule, with only compounds **1**, **2** and **5** showing moderate activity. Compound **2** (18-aminoferruginol) was the most potent.

Acetylation on the hydroxyl group at C-12 produced more potent compounds (**3** and **5** vs. **4** and **8**). However, oxygenation at C-7 to produce ketone analogues of sugiol (**11**) and methyl ester or carboxylic acid at C-18, such as compounds **8** and **9**, led to inactive compounds. Our previous study showed a similar trend with leukaemia cell lines [19].

These abietane-diterpenoid derivatives are promising sources of bioactive molecules with interesting pharmacological and drug-like properties. Table 2 data show that despite half of the compounds violating Lipinski’s rule of Log *p* < 5, some compounds like ferruginol (**1**), methyl 12-acetoxy dehydroabietate (**3**), 18-hydroxyferruginol (**7**) and sugiol (**11**) exhibit moderate activity. In contrast, some compounds that comply with Lipinski’s rule, such as methyl 18-carboxy sugiol (**8**) and 18-carboxy sugiol (**9**), show no activity. 

The most active analogue, 18-aminoferruginol (2), complies with Lipinski rules and reveals an increased polar surface area and decreased lipophilicity, which increases its drug-likeness and—according to expectations—is here associated with much lower GI50 values. It is important, however, to keep in mind that although increasing lipophilicity is considered to be an advantage in experimental models as it favours a passive entrance to the cell via diffusion through the membranes [35], it does not always translate well into favourable clinical parameters [36].

### 3.2. Introduction of the Amino Group in Position 18 Changes the Dynamics of the Apoptotic Process of the Parent Molecule in SK-MEL-28 Cells 

A mitochondrial permeability transition event usually indicates cellular apoptosis initiation, while caspase 3/7 activation signals the late apoptosis phase [25]. Ferruginol induces apoptosis in several melanoma cell lines, including SK-MEL-28 [13,16]. In our study, ferruginol triggered early depolarisation of the mitochondrial membrane in SK-MEL-28 melanoma cells, but this did not lead to caspase 3/7 activation. Only its 18-amino derivative activates caspases at later time points. Conversely, 18-aminoferruginol did not replicate its parent molecule’s depolarising effect on mitochondrial membranes but activated caspases as effectively as the reference drug paclitaxel. This seemingly counterintuitive effect aligns with emerging data suggesting that dissipation of Δψm may or may not be an early event in the apoptotic pathway, depending on the cell system under investigation and the apoptotic stimuli used [24].

The relationship between caspases and mitochondrial membrane potential is an evolving paradigm. Single-cell imaging experiments have demonstrated that the mitochondrial membrane potential depolarises after or concurrently with the release of a cyt-C-GFP fusion protein, a process described as caspase-dependent [37]. In addition to apoptosis, emerging data suggest a direct link between mitochondria and caspases in regulating non-apoptotic processes such as mitochondrial dynamics, autophagy, mitochondrial membrane permeabilisation, immune responses and differentiation [38]. Bcl-2 and Bcl-xL significantly regulate intrinsic apoptosis by protecting the mitochondrion. Previous studies on abietane diterpene derivatives have shown that even overexpression of these protective proteins does not confer resistance to abietane diterpene-induced cytotoxicity in several cancer cell lines [39]. Future research should explore the effects of such compounds on apoptosis resistance factors like Smac/DIABLO and AIF [40].

The anti-migratory and anti-invasive effects of abietane diterpenoids appear to be poorly defined. Previous research reports that abietic acid exhibits such effects in non-human B16 melanoma cells at similar concentrations [41], while tanshinone II reduces CXCL12-induced human melanoma A375 cell invasive ability and migration in a dose-dependent manner [42]. However, in our experiments, ferruginol and its derivatives did not significantly affect SK-MEL-28 cell movement in transwell models at 10 µM. Therefore, more efforts are needed to screen additional abietane diterpenes to determine their potential to inhibit cancer cell motility.

### 3.3. Effect of Ferruginol Derivatives in Other Cancer Cell Lines: Prospects

The effects of semisynthetic ferruginol and some selected analogues, including 18-aminoferruginol, have also been tested by one of the authors in four breast cancer cell lines, namely SUM149, MDA-MB231, T47D and MCF-7 [17]. Then, again, 18-aminoferruginol consistently showed better cytotoxicity than its parent molecule while keeping low cytotoxicity towards the non-tumorigenic cell line BJ. A selective effect is considered a crucial parameter for effective cancer treatment to exert high antitumor activity and minimal toxicity to normal tissues. Therefore, it is considered a good indicator of reduced side effects [43]. Notably, a ferruginol analogue containing a phthalimide group at C18 also showed increased antiproliferative activity and a selectivity index similar to 18-aminoferruginol [17]. 

Further research is needed to understand the exact nature of the improvement due to adding the amino group at C-18. This finding aligns with previous studies using slightly different parent abietane templates. For instance, dehydroabietylamine—also called leelamine—proved to be a better antitumor agent than its parent molecule, dehydroabietic acid. It has the same amino group in position 18 and targets multiple key signalling pathways in melanoma cells, including uncommon ones such as cancer cell death via inhibiting intracellular cholesterol transport [44,45,46]. This data prompted the synthesis of further derivatives—such as abietylamine—to improve these bioactivities [19,47] and protect intellectual property [48]. A significant difference between these derivatives and ferruginol is the absence of the phenol group. In our study, we have retained it as an integral part of the pharmacophore moiety or the privileged structure that we are examining in the parent ferruginol.

**Figure 9:**
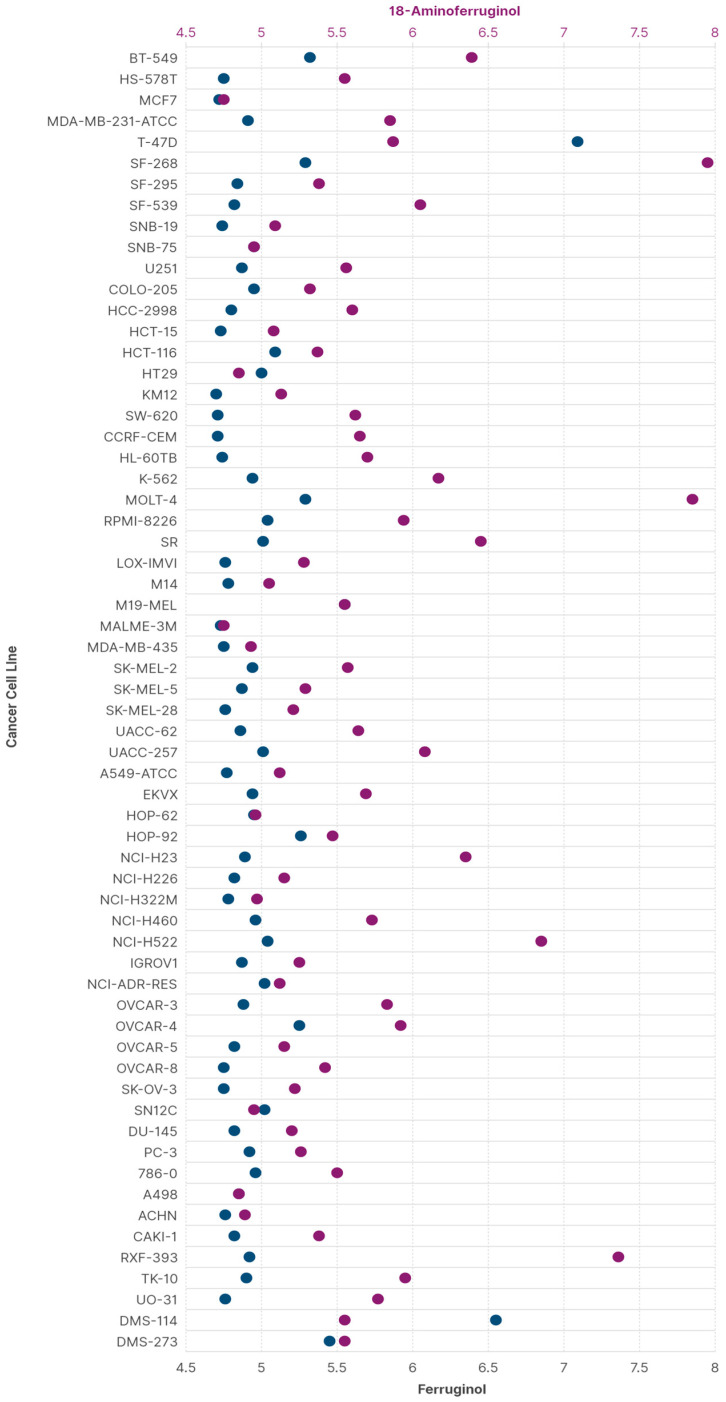
Prediction of the antiproliferative activity (−logGI_50_) of ferruginol (blue) and 18-aminoferruginol (purple) in the NCI60 panel of cancer cells (calculated by Drudit as per section Section 4.9).

These data and the above considerations for leelamine indicate that the derivatisation of abietane diterpenes in C18 is a promising way to find new, improved anticancer lead compounds. We, therefore, employed the Antiproliferative Activity Predictor (AAP) tool to calculate the GI50 values of 18-aminoferruginol and its parent molecule against the NCI60 panel. This ligand-based protocol has proven highly reliable and robust, making it particularly suitable for predicting activity against the SK-MEL-28 cell line [20]. It also uses data based on the same antiproliferative test we chose [21]. Figure 9 shows the predicted results for ferruginol and 18-aminoferruginol (−logGI_50_), with values between 4 and 6 considered more reliable [20].

The overall data reveal an improvement in ferruginol’s antiproliferative activity after amination at position 18 across most cells in the panel. Exceptions include T-47D (breast cancer), HT29 (colorectal adenocarcinoma) and HOP-62 (lung adenocarcinoma). In the first case, Gonzalez-Cardenete and coworkers showed that 18-aminoferruginol is not significantly more active (>50 µM) in the CellTitre^®^ assay than ferruginol (>100 µM), but both were considered inactive against this cell line [17].

The predicted MCF-7 (breast cancer) values align with our previous MTT test values: ferruginol 19 µM vs. 18-aminoferruginol 10 µM [17]. It is noteworthy that similar values for M19-MEL are due to a known limitation of the AAP for this particular cell line [20]. For all other melanoma cells, the improvement in activity for 18-aminoferruginol was more evident across all SK-Mel cell lines.

Our findings demonstrate that converting ferruginol into 18-aminoferruginol increases its antiproliferative activity fivefold in SK-MEL-28 cells. It also alters the apoptotic mechanism to activate caspases 3/7 without prior mitochondrial membrane depolarisation. In silico predictions align with experimental data, showing that 18-aminoferruginol is more cytotoxic to a panel of 60 cancer cells than its parent molecule. These findings encourage future broader applications.

### 3.4. Conclusions

In summary, we demonstrate here that the derivatisation of ferruginol into 18-aminoferruginol increases its antiproliferative activity five times in SK-MEL-28 cells as well as changing the apoptotic mechanism to activation of caspases 3/7 without previous depolarisation of the mitochondrial membrane. In silico predictions agree with experimental data in that 18-aminoferruginol is more cytotoxic to a panel of 60 cancer cells than its parent molecule, thus encouraging future broader applications. 

These data—and the previous reports for non-phenolic abietanes such as leelamine—suggest that derivatisation of abietane diterpenes at C18 could be a promising approach to discovering new, improved cytotoxic lead compounds.

## 4. Materials and Methods

### 4.1. Materials

Ferruginol and synthetic analogues (Figure 1) were synthesised as described previously by one of the authors (M. Gonzalez-Cardenete): compounds **1** and **2** [47] and compounds **3**–**11** [19]. The characterisation data for all tested compounds were in excellent agreement with those reported by us [19,47] and similar purity, above 95% (NMR data are available on request). All other chemicals were from Sigma-Aldrich (St. Louis, MO, USA) Sulforhodamine B, trichloroacetic acid, Trizma base, propidium iodide, Ribonuclease A, formaldehyde and crystal violet. Glacial acetic acid, ethanol and methanol were from Fisher (Leicestershire, UK). Minimum essential media (MEM), heat-inactivated fetal bovine serum (FBS), penicillin–streptomycin antibiotic, non-essential amino acids solution (NEAA), sodium pyruvate, TrypLE Express (1×, trypsin, EDTA, phenol red), phosphate-buffered saline (PBS), ReadyProbes^®^ cell viability imaging kit, trypan blue and the MitoProbe JC-1 kit were from Thermo Fisher Scientific (Waltham, MA, USA). Matrigel was from BD Bioscience (San Jose, CA, USA), Caspase-Glo^®^ 3/7 from Promega and DAPI staining from Cell Signalling Technology (Danvers, MA, USA). CytoSelect Cell Migration Assay kit was from Cell Biolab Inc (San Diego, CA, USA).

### 4.2. Cell Lines

SK-MEL-28 cell line was purchased from American Type Culture Collection (Manassas, VA, USA) and was maintained in MEM containing GlutaMAx and supplemented with 10% FBS, 1% NEEA, 1 mM sodium pyruvate and 1% penicillin–streptomycin antibiotic all from Gibco (Waltham, MA, USA) under a humidified atmosphere (Air 5% CO_2_) and at 37 °C.

### 4.3. Sulforhodamine B (SRB) Assay

This assay determined the ability of the extract to inhibit cellular growth by measuring the cell density, thereby estimating cell number. This assay was performed according to previously described methods [21]. Cells were seeded at a density of 8000 cells/well in a 96-well plate (Thermo Scientific, Waltham, MA, USA) and left overnight to attach at 37 °C. Afterwards, cells were treated with ferruginol and its derivatives for 48 h. Upon the completion of the incubation period, the cells were fixed with a trichloroacetic acid solution for one hour at 4 °C. After washing with water, the cell proteins were stained with SRB solution and left at room temperature for one hour. Then, the plate was washed four times with 1% acetic acid and flicked to remove unbound dye. Then, Tris base buffer solution was added to each well, and the absorbance was measured at 510 nm. Cell growth was calculated using the following equation:

%Cell growth = (Absorbance (sample) − Absorbance (blank))/(Absorbance (vehicle control) − Absorbance (blank)) × 100

The selectivity index (SI) has been calculated as indicated by Indrayanto, Putra and Suhud [43]:

SI = IC50 non-cancer cell/IC50 cancer cell

### 4.4. Caspase-3/7 Activity Assay

The apoptosis induced using compounds was determined by measuring the activity of caspase-3/7 using Caspase-Glo^®^ 3/ according to the manufacturer’s protocol (Promega, Madison, WI, USA). SK-MEL-28 cells were seeded in a 96-well white plate and treated with compounds or DMSO for different time points (12, 24 and 48 h). Afterwards, 100 μL of culture media of each well was transferred to a 96 white multi-well plate, and 100 μL of Caspase-Glo^®^ 3/7 reagent was added and incubated for one hour at room temperature. When the incubation period was completed, the luminescence of each sample representing the enzymatic activity of caspase was measured using a plate-reading luminometer (Tecan, Männedorf, Switzerland). The assay was performed in three independent experiments, and each sample was performed in triplicate (mean ± SD, n = 9).

### 4.5. Phase-Contrast and Fluorescence Microscopy DAPI Staining

A morphological assessment of the cells was performed to detect the cellular changes induced using the tested compounds according to a previously reported method [5]. Cells were seeded at a density of 5000 cells in 12-well plates with or without compounds/DMSO for 48 h. Treated cells with typical morphological changes of apoptosis were imaged using a phase-contrast inverted microscope (EVOS cell imaging system, Thermo Fisher Scientific, Waltham, MA, USA). 

Cells were treated in the same condition and examined with a fluorescence microscope using DAPI staining dye (Cell Signaling Technology, Danvers, MA, USA). The dyes were added and incubated at 37 °C for 15 min, and the wells were visualised using the EVOS cell imaging system. The quantitative results were obtained by capturing images from six random fields for each sample using three pictures per replicate. The fragmented nuclei were then calculated using the open-access software ImageJ v. 1.53k (National Institute of Health, LOCI, University of Wisconsin, Madison, WI, USA).

### 4.6. Determination of Mitochondrial Membrane Potential (MMP)

The mitochondrial transmembrane potential (Δψm) was determined using the fluorescent probe JC-1 (Thermo Fisher Scientific, Waltham, MA, USA). One thousand cells/well (6, 12 and 24 h) and five thousand cells/well (72 h) were seeded into 96 well, black plates with transparent bottoms and incubated. After incubation, the treated cells were washed with the warm phosphate-buffered saline (PBS) twice, JC-1 (2 μM, dye) and CCCP (50 μM, positive control) were added and further incubated for 15 min at 37 °C in a 5% CO_2_ atmosphere. The fluorescence-stained cells were measured by a fluorescence plate reader (Tecan Infinite M200, Männedorf, Switzerland) with red fluorescence in excitation (550 nm)/emission (600 nm) and green fluorescence in excitation (485 nm)/emission (535 nm). 

### 4.7. Transwell Assays

Cell migration was measured using the CytoSelect Cell Migration Assay kit (CBA-100-C, Cell Biolab Inc. (San Diego, CA, USA). Polycarbonate filters with 8-µm pore size inserts were placed in a 24-well plate. To assess invasion, the polycarbonate filters with 8-µm pore size inserts coated with a protein matrix isolated from Engelbreth-Holm-Swarm tumour cells to form a membrane layer. The coated inserts required rehydration with 300 µL warm, serum-free medium before cell seeding. To assess migration, the polycarbonate filters were not coated. SK-MEL28 cells were seeded in a serum-free medium with testing drugs onto the inserts, and the lower chambers were filled with the same serum medium containing drugs and incubated for 24 h and 48 h. Later, the remaining cells were gently removed using a cotton swab, and the inserts were washed and fixed with cold methanol. Then, the migrated cells were stained with 0.5% crystal violet for 10 min, and then inserts were washed with PBS and let air dry. Air-dried inserts were dissolved in 10% acetic acid to obtain a coloured solution. The number of the cells that migrated to the lower side of the filter was measured at 560 nm using a microtiter plate reader (Tecan Infinite^®^ M200, Männedorf, Switzerland) and presented as relative absorbance compared to control (mean ± SD) from three independent experiments.

### 4.8. Statistical Analysis

Results are expressed as mean ± standard deviation (SD) from at least three independent experiments. All data were analysed using One-way ANOVA and unpaired two-tailed Student’s *t*-test with a *p*-value of <0.05 considered significant to find the statistical significance between treated groups and controls using InStat v.3 (GraphPad, San Diego, CA, USA).

### 4.9. In Silico Prediction of the Antiproliferative Activity

The DRUDIT online 1.0 (STEBICEF-University of Palermo, Italy) service runs on four servers that are automatically selected according to the number of jobs and online availability. Each server can support up to 10 simultaneous jobs while the exceeding jobs are placed in a queue. DRUDIT consists of several software modules implemented in C and JAVA and running on MacOS Mojave. The input structures were drawn directly in the web interface. The MOLDESTO (molecular descriptor tool), implemented in DRUDIT, calculates more than 1000 molecular descriptors (1D, 2D and 3D) for each input structure [49].

The MOLDESTO file for each molecule is then submitted to the AAP tool as described by the authors [20]. It comprises the fingerprint (FP) and cell line (CL) modules, which cooperate simultaneously to assign the predicted −logGI_50_ values to each input structure. In each module, the performed calculation is dynamic; indeed, it can be modulated by appropriately tuning the values of the available parameters. The FP module parameters are a choice of biological activity, such as GI_50_, TGI, LC_50_, or G%, but in this work, only the first one was considered. The N (-b), the best number of the dynamically selected molecular descriptors; Z (-m), the number of descriptors for which |v − m|/m < <value> applies (v: descriptor value, m: target mean) were set at ½ Max value to allow for an equal bias to all cells in the panel and the Gaussian smoothing function used was set in “c mode”.

## Figures and Tables

**Table 1:** Estimation of the selectivity index (SI) for the active compounds.

Compound	SK-MEL-28	Fibroblasts	SI
**1**	47.5 µM	55 µM ^a^	≅1.2
**2**	9.8 µM	75 µM ^a^	≅7.7
Paclitaxel	10 nM	2.61 nM ^b^	≅0.3

(^a^) BJ cells [17]; (^b^) MCR-5 cells [22].

**Table 2:** Estimation of molecular properties ^(a)^ (drug-likeness) using Molinspiration online software ^(b)^ for compounds **1**–**11**.

Compound	log P	MW	n-HBA	n-HBD	TPSA	Lipinski’s Violation
**1**	6.41	286.46	1	1	20.23	1
**2**	4.67	301.47	2	3	46.25	0
**3**	5.29	372.50	4	0	52.61	1
**4**	5.73	330.47	3	1	46.53	1
**5**	4.39	386.49	5	0	69.68	0
**6**	5.11	316.44	3	2	57.53	1
**7**	5.24	302.46	2	2	40.46	1
**8**	4.83	344.45	4	1	63.60	0
**9**	4.21	330.42	4	2	74.60	0
**10**	4.33	316.44	3	2	57.53	0
**11**	5.51	300.44	2	1	37.30	1
Rule of five	not > 5	<500	not > 10	not > 5		1 violation allowed

^(a)^ P = partition coefficient; MW = Molecular weight; n-HBA = number of hydrogen bond accepting groups; n-HBD = number of hydrogen bond donating groups; TPSA = Total polar surface area. ^(b)^ Molinspiration Cheminformatics software (Molinspiration, Slovensky Grob, Slovak Republic, 2023, https://www.molinspiration.com, accessed on 8 September 2023).

## Data Availability

The data presented in this study are available on request from the corresponding author. The data are not publicly available until the first author’s Ph.D. dissertation is published.
